# The Neurolinguistics of the Heart

**DOI:** 10.5935/abc.20190218

**Published:** 2019-10

**Authors:** Esteban Wisnivesky Rivarola, Mauricio Scanavacca

**Affiliations:** 1 Universidade de São Paulo - Faculdade de Medicina Hospital das ClÍnicas -Instituto do Coração - Unidade de Arritmia, São Paulo, SP - Brazil; 2 Universidade de São Paulo - Instituto do Coração - Unidade Clínica de Arritmia, São Paulo, SP - Brazil

**Keywords:** Heart Failure;Gini Coefficient, Stress, Psychological, Action Spectrum, Sympathetic Nervous System, Parasympathetic Nervous System, Neurolinguistic Programming

The association between mental stress and cardiac function has always been of major interest. In the 19^th^ century, Claude Bernard, the father of evidence-based medicine, first acknowledged the vagus nerve as a structural and functional link connecting the heart and the brain. Nowadays, stress is considered one of the most significant health problems in modern society, related to the physiopathology of psychiatric, metabolic and cardiovascular diseases, and the search for its biomarkers remains a challenging task for researchers and clinicians.

But what is stress? How can we define it, before we tackle it?

From a phylogenetic point of view, the perception of threat and safety is the core element implicated in mental events related to stress. This threat appraisal works as a trigger of complex neurological processes leading to adaptive adjustments of heart rate, cardiac contractility and vascular resistance that follows sympathetic tone enhancement and parasympathetic withdrawal, and ultimately result in the survival of the individual and of the species. In summary, the heart and the brain are in constant communication in order to keep us from danger.

Although several physiological mechanisms are known to take part in this elaborate neurological circuitry, the autonomic nervous system is, undisputedly, the protagonist. The study "Introduction of Application of Gini Coefficient to Heart Rate Variability Spectrum for Mental Stress Evaluation",^1^ identified an increase in the low frequency spectral power and in total spectral inequalities (using the Gini coefficient) during a cognitive mental challenge (arithmetic exercise). Interestingly, the 0.1Hz band expression, frequently associated with the arterial baroreflex activation, was significantly increased. Authors therefore proposed these indexes as biomarkers of stress and implicated baroreflex hyperactivity in its psychophysiology.

To the extent that we assume autonomic tonus changes as a reliable sign of mental stress, the heart rate variability (HRV) may indeed provide some interesting information. However, a careful appreciation of the involved neural structures may yield an insight into a sophisticated system and indicate that a reductionist interpretation of the autonomic response pattern may turn out to be misleading.

Observe the peripheric autonomic nervous pathways carefully. Its extrinsic components, mainly comprised by the vagus nerve and the numerous afferent and efferent nerves of the sophisticated sympathetic thoracic chain, are responsible to carry, through the central nervous system and back to the heart, sympathetic and parasympathetic information from baro and chemoreceptors, generating balanced responses that maintain internal homeostasis. Take into consideration that most of these structures are bimodal, with both vagal and sympathetic inputs.^2^ Close to the epicardial surface, this innervation resolves into the intrinsic system, consisting of a dense net of thousands of neural cells and hundreds of epicardial ganglia, plentifully located in the atrial surface.^3,4^ Cardiac ganglia work as integrative centers, where efferent data can be modulated, so the whole system can flexibly respond to a wide range of stimuli.^5^ This modulation, this capacity to provide adaptive control over the periphery, is the hallmark of the autonomic nervous system.

Intrinsic and extrinsic systems are connected to the central nervous system. Here is where things start getting tricky. By using PET Scan and MRI, a series of neuroimaging studies^6,7^ describe a *central autonomic network,*^7^ containing cortical and subcortical areas, through which the brain controls visceromotor functions and goal-directed behavior. The network includes prefrontal cortices, the central nucleus of the amygdala, the paraventricular nucleus of the hypothalamus, the parabrachial nucleus, the nucleus of the solitary tract, and the nucleus ambiguous, among others. All these components are reciprocally interconnected, and the interplay of these inputs provides flexible adjustments. The system essentially operates as a continuous integration of concepts such as “self” and “danger” with external perceptions and memory into Gestalt representations, generating likely responses.

After appraising a potential threat, a primitive quick mental stress reaction arises from the amygdala. The reaction to uncertainty or danger is a relatively simple sympathoexcitatory state known as “fight or flight”, that in its pristine form results in a rather predictable HR increment. However, this initial perception often gives way to more elaborate mental interpretations as certain cortical areas action unfolds. The frontal cortex (FC), and medial preFC in particular, has a significant role by activating GABAergic pathways exerting inhibitory control over an activated amygdala.^7^ The more abstract the stressful event, the more important and modulated the inhibition of subcortical cardioacceleratory circuits are, meaning that all these neural structures can be differentially recruited depending on the nature of the challenge, creating context-specific response patterns.

Complex tasks requiring cognitive functions, such as arithmetic, online processing and manipulation of information are highly dependent on this reciprocal downregulation between the cortex and the amygdala.^8^ The resultant HRV takes so many variables into account that defining specific spectral bands as reliable biomarkers, assuming a mechanistic causality, seems unlikely, especially so when it comes to abstract stressful contexts, such as expectations of future outcomes, emotional conditions and representation of economic values.^7^

To illustrate this limitation, in a very simplified way, imagine you are about to take a math test. At first, the challenge may disturb you, being felt as “danger”, and so the amygdala nucleus promptly triggers a vagal withdraw (affecting both high and low frequency spectral powers) and a sympathoexcitatory reflex (modifying low frequency spectra). A few minutes later you realize you can handle it and start feeling confident. It is just going to take a little focus. A more accurate Gestalt representation has now been reached. By activating a GABA pathway, three main different areas of your preFC engage: 1-posterior and dorsal region of the rostral preFC (linked to cognitive functions), 2-dorso medial preFC (reliably related to social cognition) and finally 3- medial-orbitoFC and anterior ventral preFC (associated with autonomic aspects of emotional contexts and to “reward and punishment”).^6,7^

Exerting a balanced inhibitory control over the amygdala through an integrated Central Autonomic Network, these anatomic structures guide goal-directed behavior and adaptability and ultimately dictates the amount of acetylcholine and norepinephrine to be released from the post-ganglionic fibers close to the sinus node, reshaping HRV spectra once again. Keep in mind that genetic background and cognitive performance may influence all these processes significantly.^7^

What is the value of HRV on assessing stress, then? As of 1965, Hon and Lee^9^ had already identified inter-beat interval patterns preceding severe fetal distress even before a perceivable change in fetal heart rate. Undoubtedly, HRV has been proven to be an essential index of adaptability of the organism, and therefore, extensively studied under a wide range of stressful stimuli. Conflicting results, however, suggest distinct reactions to different forms of stress. While some authors demonstrated a global increase in HRV after exposure to noise,^10,11^ public speech tasks^12^ and sustained attention,^13^ others found a global HRV reduction during memory^14^ and cognitive tasks.^15^ Emotional conditions have been proven to correlate with high-frequency band reduction by some authors,^16^ and yet to be neutral by others.^12^

We are far from relying on surrogate endpoints to understand the different parts of the link between heart and brain. Even by using much more sophisticated techniques, such as regional cerebral blood flow neuroimaging, we are still scratching the surface of this intricate physiology. HRV could be an interesting tool for detecting general features of the stress response, although unreliable for distinguishing its complex mechanisms.
